# A case study: Re-evaluating the biological risk following a processing aid change on a marketed cardiovascular implant device

**DOI:** 10.3389/fmedt.2022.1006984

**Published:** 2022-11-28

**Authors:** Frances K. Hsia, Alessia Stornetta, Nicole V. Soucy

**Affiliations:** Global Toxicology and Biocompatibility, Preclinical Sciences, Boston Scientific Corporation, Arden Hills, MN, United States

**Keywords:** medical device, biological risk evaluation, toxicological risk assessment, extractable, leachable, threshold of toxicological concern, ISO 10993

## Abstract

Per ISO 10993-1:2018, a processing change to a medical device requires re-evaluation of biological risk. Here, we present the biological evaluation of a marketed cardiovascular implant following a detergent formulation change. This change was initially assessed through a qualitative toxicological risk assessment based on the fully disclosed detergent formulation and a limited panel of biological testing. The conclusion was that the new detergent did not impact the biological safety of the device. This assessment was rejected during regulatory review, and extractables and leachables under exhaustive extraction conditions were then evaluated for devices processed with new versus original detergent. New extractables were present at low levels (2–65 µg/device), and a toxicological risk assessment concluded no concern. The regulatory agency responded requesting additional biological testing to evaluate local effects, further characterization of compounds with a “tentative” identification, and leachable data to support clinically relevant exposure estimates. All additional data was collected per the agency request. Still, the conclusion, considering all data, was unchanged, suggesting the extensive chemical characterization and repeat biological testing unnecessary, especially considering animal use. This case study highlights the recent shift in regulatory expectations around chemical characterization and questions the value of additional biological testing when faced with low extractable levels of low toxicity concern. It also demonstrates the need to hold to key portions of the ISO 10993 risk management framework to avoid excessive burden on medical device development when there is little to no determined risk to patient safety.

## Introduction

Medical devices are used in the prevention, diagnosis, and/or treatment of disease and provide benefits that improve patients’ lives. To protect patients from potential biological risks arising from the use of medical devices, the safety of each device must be assessed in accordance with the ISO 10993 standard series. ISO 10993-1 *Biological evaluation of medical devices—Part 1: Evaluation and testing within a risk management process* provides guidance on how to perform a systematic biological safety evaluation for a medical device (1). The standard also aims to minimize animal testing by giving preference to *in vitro* models and chemical characterization.

Manufacturing of medical devices occurs under Good Manufacturing Practice and Quality System to ensure that products consistently meet applicable requirements and specifications. Modifications to a medical device can be driven by a number of factors including changes in design, process, material, or supplier. When modifications are proposed for a marketed device, the biological risk shall be re-evaluated following the systematic approach and general principles outlined in ISO 10993-1 (1). The objective of this perspective article is to present a case study wherein the biological risk of a marketed device required re-evaluation following a change to a processing aid. In this case study, we show that the additional assessments requested by the regulatory agency were unnecessary and did not demonstrate any added risk or concern to patient safety. We outline an alternative approach to address such a change in the future that is more aligned with the “least-burdensome” concept inherent to medical device regulatory frameworks.

## Background

The device under assessment was a metallic cardiovascular device used in adults, classified per ISO 10993-1 as an implant with long-term (>30 days) contact with blood (1). The device has been globally marketed for more than 5 years. During the final device cleaning process, a detergent commonly used in the medical device industry is used to remove residual manufacturing and processing aids. Following use of the detergent, the implant is rinsed three times with distilled water to ensure that the device is free of residuals. The detergent was re-formulated in 2016, replacing a carcinogenic substance and two other components in the formulation with alternative, safer substances. This change triggered a re-evaluation of the biological risk of the device according to the principles and guidelines of ISO 10993-1.

**Figure 1:**
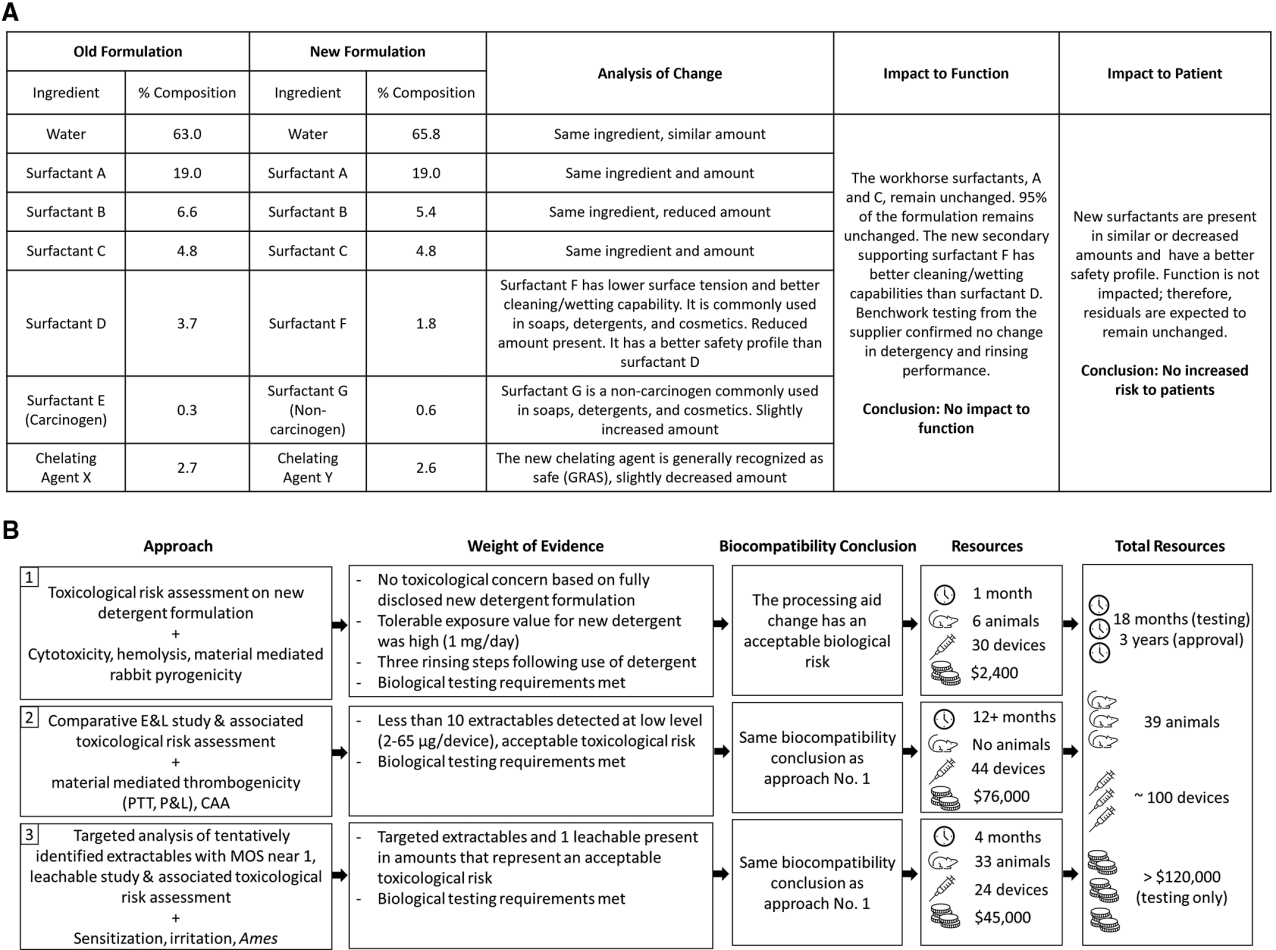
(**A**) Comparison of new and old detergent formulation and analysis of impact to function and patient risk; (**B**) path to achieving biocompatibility regulatory approval following a processing aid change used in the manufacturing of an implant medical device.

Since the complete formulation of the detergent was disclosed, the approach initially taken to re-evaluate the biological risk of the device was a qualitative toxicological risk assessment (TRA) based on toxicological literature review of each ingredient. The TRA was supported by a limited panel of biological tests that consisted of cytotoxicity, hemolysis, and material-mediated pyrogenicity testing on final devices processed with the new detergent. Each biological test met test-specific requirements. Key to the overall assessment was that the major constituents of the new detergent formulation remained unchanged and all three new replacement chemicals, representing 5% of the total composition, have low intrinsic hazard (eye/skin irritants only) and are commonly used in soaps, detergents, and cosmetics. The new formulation removed a carcinogenic surfactant and replaced it with a surfactant with lower surface tension, and better cleaning/wetting capabilities. The chelating agent was also replaced with one that is generally recognized as safe (GRAS) and used in therapeutics (Figure 1A). Considering that the manufacturing process included three rinsing steps, the level of residual detergent was expected to be low, and similar to levels previously detected through analytical work completed on the device washed with the original detergent. In addition, given that the parenteral tolerable exposure on the new formulation was 1 mg/day (derived from an acceptable oral daily exposure of 10 mg/day), potential residuals from the new detergent were assessed to be toxicologically insignificant.

Based on the evaluation described above, it was concluded that the changes to the detergent formulation would have no impact on the biological risk of the device (Figure 1B). However, when reviewed by a regulatory agency in 2018, the totality of evidence was considered insufficient. The regulatory agency was concerned that new or increased chemical residuals could be present on the device either from the new detergent, or because the new detergent would not be as efficient at cleaning the device. Therefore, the agency recommended assessing acute/subacute/subchronic/chronic toxicity, genotoxicity, and carcinogenicity endpoints *via* a comparative extractable and leachable (E&L) study and TRA, using devices processed with the new and original detergent. In addition, the agency suggested that if new chemicals or higher amounts of chemicals were detected on the device processed with the new detergent from the E&L study and no substance specific toxicological data was available on those chemicals, then biological testing of the final device to assess irritation, sensitization, material-mediated thrombogenicity and complement activation was recommended.

## Comparative E&L study and toxicological risk assessment

Following initial agency feedback, a comparative E&L study designed to evaluate chemical equivalence between devices processed with the new and the original detergent formulation was reviewed and approved by the regulatory agency in 2019 prior to study initiation. The initial study plan was to include multiple devices into a single exhaustive extraction for each solvent, with the justification that control processes to address variation within and across product lots were in place. Additionally, multiple devices per extraction solvent would consider any unexpected variation. This plan was not accepted by the regulatory agency. The concern was that the single extraction would not capture the variability in the extraction process and analytical method. Therefore, extractions were performed in triplicate and analyzed separately. For gravimetric analysis, the test articles were extracted at a ratio of 6 cm^2^/ml in purified water, ethanol, and hexane at 50 ± 2°C in 24-h increments, in accordance to ISO 10993-12 and ISO 10993-18 (2, 3). Water was used for the polar extraction for gravimetric analysis to avoid the background commonly observed during non-volatile residue (NVR) analysis from salt in the saline.

Exhaustive extraction, as defined in ISO 10993-18, was achieved after the first round of extraction in water and hexane, and after the second round of extraction in ethanol. The average NVR mass observed per device was <0.049 mg (water), 0.237 mg (ethanol) and 0.137 mg (hexane). For chemical characterization, the test articles were extracted at a ratio of 6 cm^2^/ml in 0.9% saline, ethanol, and hexane at 50 ± 2°C for the duration determined by gravimetric analysis (24 h for 0.9% saline and hexane; 48 h for ethanol). The triplicate extracts were analyzed by Headspace Gas Chromatography Mass Spectrometry (HS-GCMS), Quadrupole Time of Flight Gas Chromatography Mass Spectrometry (QTOF-GCMS), and Quadrupole Time of Flight Liquid Chromatography Mass Spectrometry (QTOF-LCMS); the saline extract was also analyzed by Inductively Coupled Plasma Mass Spectrometry (ICP-MS). GCMS and Ultrahigh Performance Liquid Chromatography-Ultraviolet-Charged Aerosol Detection (UHPLC-CAD-UV) were used for relative quantification of identified compounds in the extracts. The instrument detection limit was 1 µg/device.

No differences in volatile organic compounds were observed by HS-GCMS in any solvent between devices processed with new vs. original detergent. No difference was observed by QTOF-GCMS, QTOF-LCMS, or ICP-MS in the saline extract between devices processed with new vs. original detergent. Six individual extractables and two groups of related compounds were identified by QTOF-GCMS or QTOF-LCMS in the ethanol and/or hexane extracts as unique to or ≥3X elevated^1^ in the device processed with new vs. original detergent. The maximum total amount of any compound or group of related compounds identified was 65 µg/device, see Table 1.

**Table 1:** Risk characterization of extractables and leachables from devices processed using the new detergent formulation.

Chemical name/CAS RN (confidence level)	TE or [TTC] (µg/day)	Exhaustive E&L study		Simulated-use study		
		Worst-case exposure (µg/day)^c^	Worst-case MOS	Clinical exposure (µg/day)		Clinical MOS
				Day 1	Day 2	
4-Oxo-β-isodamascol/NA (tentative)	[1.5]	4.8	0.3	NA		NA
Ethoxy (methyl)phenylsilanol^a^/NA (tentative)	[90]	4.8	19	ND	ND	>60
Diethyl terephthalate/636-09-9 (confirmed)	45,000	13.9	3,237	ND	ND	>30,000
2,4,6-Trimethyl-2,4,6-triphenylcyclotrisiloxane/546-45-2 (confirmed)	[90]	3.0	30	ND	ND	>60
N-Pentyl-1-octanamine/6835-13-8 (tentative)	[90]	64.9	1.4	NA		NA
N-Methyldodecylamine^b^/7311-30-0 (confirmed)	[90]	64.9	1.4	ND	ND	>60
4-Tridecylbenzenesulfonic acid/25496-01-9 (tentative)	15,000	13	1,154	2.9	ND	5,172
Fatty amides/NA (tentative)	60,000	2.4	25,000	ND	ND	>40,000
Oxidized TBPP (Irgafos 168)/95906-11-9 (confirmed)	3,480	12.9	270	ND	ND	>2,320
Siloxane related and dimethylsiloxane-co-methlylphenylsiloxane oligomer related/NA (tentative)	17,460	51.5	339	ND	ND	>11,640

NA, not applicable; ND, not detected above AET; TE, tolerable exposure; TTC, threshold of toxicological concern; MOS, margin of safety.

^a^ Revised identification for 4-oxo-β-isodamascol.

^b^ Revised identification for N-pentyl-1-octanamine.

^c^ Based on the highly conservative assumption that extracted amounts are released daily over the entire lifetime of the device.

Toxicological information was available for five substances (or for a structural analogue for read-across). The risk assessment performed in accordance with ISO 10993-17 (4) concluded that none of these five compounds were of toxicological concern (Table 1). A threshold of toxicological concern (TTC) approach was utilized for the remaining three substances (5). One tentatively^2^ reported substance, 4-Oxo-β-isodamascol, was identified as a potential mutagen and had a margin of safety (MOS) of 25 when assessed using a TTC of 120 µg/day, supporting an acceptable toxicological risk. The use of less-than-lifetime TTC of 120 µg/day was appropriate to address residual processing aids which are expected to have acute exposure. Furthermore, 4-Oxo-β-isodamascol was unlikely to pose a sensitization risk, as the total estimated exposure was less than the sensitization TTC of 5 µg/day for a parenteral extractable/leachable (6). When assessed against the long-term (>30 days) TTC of 1.5 µg/day as preferred worst-case approach by the regulatory agency, the MOS was <1 (Table 1). For context, the assumption that the maximum extracted amount of 4.8 µg is released daily over the entire lifetime of the device is extremely conservative, if not implausible, as it violates basic principles of mass balance in overestimating the potential risk from exposure to extractables following initial implantation of the device.

All extractables were also assessed to pose no irritation, sensitization, or unacceptable toxicological risk. Together, the comparative E&L study and additional hemocompatibility test data (complement activation assay (CAA), partial thromboplastin time (PTT), platelet and leukocyte counts) confirmed that the change in the detergent formulation had no impact on the biocompatibility of the device (Figure 1B). However, once again the scientific rationales provided were not considered sufficient. For chemicals with MOS near 1, the regulatory agency requested additional rationale to support that the identification was reliable (in the form of *a priori* knowledge or analytical confirmation).

## Further characterization, simulated use study and additional biocompatibility testing for local effects

Given the regulatory feedback, a targeted GCMS and LCMS analysis was completed utilizing reference standards to further elucidate the identity of two tentatively identified compounds with MOS near 1. This follow-up analysis was conducted on both reserve extracts from the initial comparative E&L study and newly prepared ethanol extracts generated using conditions identical to those of the initial E&L study. In the targeted GCMS analysis of the reserve ethanol extract, the compound previously tentatively identified as 4-Oxo-β-isodamascol was now tentatively identified as ethoxy(methyl)phenylsilanol. The analytical laboratory confirmed the ethoxy(methyl)phenylsilanol to be an alcoholysis degradation product of triphenyltrimethyl-cyclotrisiloxane, which was detected in the original analysis. Ethoxy(methyl)phenylsilanol was not detected in the newly prepared ethanol extract. The substance was predicted to be non-mutagenic by an expert rule-based (Toxtree) and a statistical-based computational model (VEGA) (7, 8). In addition, a bacterial reverse mutation test (Ames assay) conducted on the device processed with the new detergent demonstrated that the device was non-mutagenic and had no carcinogenic potential. When ethoxy(methyl)phenylsilanol was assessed using the appropriate TTC of 90 µg/day for a non-carcinogen (Cramer Class III) (5), the resulting MOS was 19. The other compound with a MOS near 1 that was previously tentatively identified by LCMS as N-pentyl-1-octanamine was detected in both the reserve and new ethanol extracts and was confirmed as N-methyldodecylamine. This compound was potentially related to a surfactant used in the new detergent formulation (Table 1). The change in identification of these two compounds and absence of one of them in the newly prepared extract highlights a limitation of these analytical methods in reproducing data when dealing with low level extractables.

Additional requests for supporting data triggered biological testing to evaluate local effects not previously evaluated by testing (i.e., irritation and sensitization) and a leachable study to further refine daily exposure estimates. The biological tests confirmed there was no irritation or sensitization concern for devices processed with the new detergent formulation. To simulate clinical use conditions in the leachable study, devices processed with new detergent were extracted in 40% ethanol/water at 37°C with agitation at 50 rpm for 24 h and then re-extracted for another 24 h. The use of 40% ethanol/water was based on ISO 10993-18:2020, Annex D Table D.5, where a 40% (by volume) mixture of ethanol/water is considered an appropriate surrogate for blood (3). The resulting extracts were analyzed by QTOF-GCMS and QTOF-LCMS and targeted analysis was completed for ethoxy(methyl)phenylsilanol and N-methyldodecylamine, as well as other extractables that were previously only tentatively identified. Of all the compounds specifically targeted in the leachable study, only one substance was detected above the analytical evaluation threshold (AET) calculated using a dose-based threshold (DBT) of 1.5 µg/day. This substance was 4-tridecylbenzenesulfonic acid, which was detected at 2.9 µg/device in the first 24-h extraction, but not in the second 24-h extraction (Table 1). The additional targeted GCMS and LCMS analysis, leachable study, updated TRA, and irritation and sensitization test results supported the final approval of the change in 2021 (Figure 1B).

## Discussion

The biocompatibility of medical devices is evaluated following principles and guidelines reported in the ISO 10993 standard series—*Biological evaluation of medical devices*. Recent updates have evolved the standards into a framework that focuses on reducing unnecessary testing, including those utilizing animals, and instead on promoting *in vitro* testing, as well as on advocating for the least burdensome concept while still prioritizing patient safety. This case study illustrates the re-evaluation of biological risk for a marketed implant device following a processing aid formulation change. The extent of the data required by the regulatory agency to conclude that this minor change did not impact the biological safety of the device highlights the increased level of scrutiny being applied to biocompatibility assessments. It also demonstrates that despite the intent of the ISO 10993 series—which attempts to rely more on sound scientific rationales and limit unnecessary testing, certain regulatory agencies will still require extensive chemical characterization and biological test data to support even minor changes in device manufacturing. The lack of acceptance of the initial toxicological assessment and the resulting increased testing requirements presented a burden that is arguably not commensurate with the risk presented by the change.

The authors believe that the qualitative TRA, including the scientific evidence of improved cleaning/wetting capabilities, and confirmatory cytotoxicity and hemolysis tests adequately supported this specific formulation change to a processing aid with full compositional disclosure. This initial biological evaluation answered concerns as to residual risk and concluded that there was no impact on biocompatibility. The regulatory agency's concern for increased chemical residuals drove additional chemical and biological testing that was time-consuming, expensive, and involved the use of additional animals in arguable unnecessary testing. In order to assess this change in line with the regulatory expectations, three E&L studies were performed, and all local biocompatibility assays as well as *in vitro* hemocompatibility and genotoxicity assays were repeated. This tremendous effort involved a 3-year long approval process, approximately 100 devices, and unnecessary animal use (Figure 1B). It is our opinion that none of this rework aligns with a least burdensome approach or with the current framework of the ISO 10993 standard series.

The resulting strategy to assess this detergent change did not follow a stepwise approach as outlined in ISO 10993. Instead, an overly conservative approach was required that included several rounds of biological and analytical testing that provided no added value above and beyond the initial assessment. More broadly, this case study is not an isolated instance. In fact, in recent years, the stringent demand for conservatism in toxicological risk assessment has increased, as have E&L expectations. When combined with inconsistency in acceptable approach from different global regulatory agencies, this has created tremendous challenges for medical device manufacturers to navigate. Specifically for E&L work, industry would benefit from more specific guidance regarding extraction conditions (solvents for simulated use, extraction duration) and method suitability (e.g., how to derive uncertainty factors for the AET calculation).

When selecting the TTC as the DBT per ISO/TS 21726 (5), the nature of the potential extractables and their exposure duration should be considered instead of the device's contact duration. As shown in the simulated-use study, processing aid chemicals are surface residuals expected to be released in the first 24 h; therefore, a TTC value of 120 μg/day would have been more appropriate as the DBT in the calculation of the AET. Comparative E&L studies conducted using a 1.5 μg/day DBT are likely to result in differences that are irrelevant to the risk evaluation. Although the use of 120 μg/day as the DBT for long-term contact devices is not yet recognized by regulatory agencies, it has scientific validity. In fact, Kennedy and Spinti in 2021 (9) demonstrated that an AET based on a TTC of 120 µg/day is protective and practical for all medical devices, regardless of contact duration. In this case study, if such an approach were applied, a comparative E&L study using a DBT of 120 µg/day would have resulted in equivalent profiles, and additional testing could have been avoided. Alternatively, only the clinically-relevant leachable profiles of each device could have been compared, bypassing the reporting of irrelevant extractables artificially created from the exhaustive extraction conditions.

It is also important to recognize that a TRA should not only be limited to assessing systemic toxicity, reproductive/developmental toxicity, genotoxicity, and carcinogenicity endpoints. The authors challenge the assumption that irritation and sensitization can only be addressed through biological testing, since irritation is related to local concentration. In a recent review article by Parris et al., the authors concluded that it is highly unlikely for low concentrations of E&L compounds, migrated into parenteral drug products, to result in an irritating effect. In addition, even for substances classified as strong or extreme skin sensitizers, the likelihood for induction of sensitization following parenteral exposure at 5 µg/day is considered negligible (10). Therefore, the justification provided in the TRA should have been considered sufficient to conclude that microgram levels of tentatively identified extractables detected in extracts generated under exhaustive conditions are not expected to result in an irritation or sensitization effect to patients receiving the implant processed with the new detergent. The additional irritation and sensitization testing in animals was therefore unjustified.

In conclusion, we emphasize that biocompatibility evaluation is a challenging area for both medical device manufacturers and regulatory agencies to navigate. The authors hope that this perspective article can be a starting point for discussions to improve how unavoidable changes to marketed devices can be assessed in an efficient and sustainable manner, while keeping patients safe as they receive treatment with these lifesaving devices.

## Data Availability

The original contributions presented in the study are included in the article further inquiries can be directed to the corresponding author/s.

## Footnotes

^1^The threshold of ≥3X relates to general guidance provided by ICH Harmonised Tripartite Guideline—Validation of Analytical Procedures: Text and Methodology Q2 (R1) and others concerning signal to noise and instrumental detection limits. A value of 3:1 signal to noise represents a consensus value for method limit of detection (LOD) when determined based on instrumental noise. Based on data collected for many E&L studies, the large majority of compounds observed are at low concentrations near the detection limit of the instrument. Therefore, the majority of compounds detected are competing with the noise level of the instrument. Those compounds which are found to contain a 3X difference in observed signal intensity can be confidently concluded to be unique to the sample under study.

^2^Confidence level of identified compounds was based on guidance provided by USP<1663> “Assessment of extractables associated with pharmaceutical packaging/delivery systems”, a U.S. Pharmacopeia (USP) reference standard.

## References

[1] ISO 10993-1. Biological evaluation of medical devices - part 1: evaluation and testing within a risk management process. Geneva, Switzerland: International Standards Organization (2018).

[2] ISO 10993-12. Biological evaluation of medical devices - part 12: sample preparation and reference materials. Geneva, Switzerland: International Standards Organization (2012).

[3] ISO 10993-18. Biological evaluation of medical devices - part 18: chemical characterization of medical device materials within a risk management process. Geneva, Switzerland: International Standards Organization (2020).

[4] ISO 10993-17. Biological evaluation of medical devices - part 17: establishment of allowable limits for leachable substances. Geneva, Switzerland: International Standards Organization (2002).

[5] ISO/TS 21726. Biological evaluation of medical devices – application of the threshold of toxicological concern (TTC) for assessing biocompatibility of medical device constituents (2019).

[6] Ball D Blanchard J Jacobson-Kram D McClellan RO McGovern T Norwood DL Development of safety qualification thresholds and their use in orally inhaled and nasal drug product evaluation. Toxicol Sci. (2007) 97(2):226–36. 10.1093/toxsci/kfm058 17369604

[7] LECT. VEGA QSAR non-interactive client, version 1.1.5-b36. Laboratory of environmental chemistry and toxicology. Milan, Italy (2020). Available at: https://www.vegahub.eu/portfolio-item/vega-qsar/.

[8] Jeliazkova N Martinov M. Toxtree - estimation of toxic hazard - a decision tree approach v3.1.0. 2018.

[9] Kennedy TA Spinti MJ. How sensitive does chemical characterization of medical devices need to be? Calibration of analytical evaluation thresholds with the carcinogenic potency database. Regul Toxicol Pharmacol. (2021) 122:104899. 10.1016/j.yrtph.2021.104899 33621616

[10] Parris P Whelan G Burild A Whritenour J Bruen U Bercu J Framework for sensitization assessment of extractables and leachables in pharmaceuticals. Crit Rev Toxicol. (2022) 52(2):125–38. 10.1080/10408444.2022.2065966 35703156

[11] Hsia FK Soucy N Dusenbury K Schulz W. A case study: re-evaluating biological risks involving a processing aid change on a marketed long-term implant. Abstract Number: 3462. In: Toxicologist (2022).

